# Integration of parallel metabolomics and transcriptomics reveals metabolic patterns in porcine oocytes during maturation

**DOI:** 10.3389/fendo.2023.1131256

**Published:** 2023-02-01

**Authors:** Ming Gao, Minjian Chen, Qiuzhen Chen, Shuai Zhu, Hengjie Wang, Weizheng Yang, Xi Wang, Qiang Wang, Ling Gu

**Affiliations:** ^1^ College of Animal Science & Technology, Nanjing Agricultural University, Nanjing, China; ^2^ Key Laboratory of Modern Toxicology of Ministry of Education, School of Public Health, Nanjing Medical University, Nanjing, China; ^3^ State Key Laboratory of Reproductive Medicine, Suzhou Municipal Hospital, Nanjing Medical University, Nanjing, China

**Keywords:** oocyte, energy metabolism, metabolomics, transcriptomics, reproduction

## Abstract

Well-controlled metabolism is the prerequisite for optimal oocyte development. To date, numerous studies have focused mainly on the utilization of exogenous substrates by oocytes, whereas the underlying mechanism of intrinsic regulation during meiotic maturation is less characterized. Herein, we performed an integrated analysis of parallel metabolomics and transcriptomics by isolating porcine oocytes at three time points, cooperatively depicting the global picture of the metabolic patterns during maturation. In particular, we identified the novel metabolic features during porcine oocyte meiosis, such as the fall in bile acids, the active one-carbon metabolism and a progressive decline in nucleotide metabolism. Collectively, the current study not only provides a comprehensive multiple omics data resource, but also may facilitate the discovery of molecular biomarkers that could be used to predict and improve oocyte quality.

## 1 Introduction

There is a growing awareness that oocyte quality, which is tightly correlated with meiosis, is a key limiting factor in female fertility. In most mammals, oocytes initiate the early stages of meiosis during fetal development and remain arrested at the diplotene stage (germinal vesicle, GV) of meiotic prophase I around the birth (1). At puberty, stimulated by preovulatory endogenous LH surge, fully grown oocytes reinitiate meiosis, characterized by germinal vesicle breakdown (GVBD). Accompanying with spindle organization and chromosomes alignment, oocytes proceed through the meiosis I (MI) and remain arrested in metaphase II (MII) until fertilization occurs (2). The process from GV to MII is called ‘oocyte maturation’, which involves the integration of complex nuclear and cytoplasmic changes that are a prerequisite for successful fertilization and subsequent embryo development (3). A variety of metabolites and metabolism-related enzymes experience precisely programmed changes during oocyte meiotic maturation, playing critical roles in multiple cellular events. Oocyte quality reflects intrinsic developmental potential of oocytes, however, what constitutes oocyte quality or the mechanisms governing it deserve further investigation.

Reproduction is related with metabolic state of organism in female mammal animals. Well-balanced and timed energy metabolism is critical for optimal development of oocytes (4). Emerging evidence has indicated that maternal metabolic disorders such as obesity (5, 6), diabetes (7) and polycystic ovary syndrome (PCOS) (8) which may contribute to meiotic defects, organelle dysfunction and epigenetic alteration, have a major adverse effect on oocyte maturation and early embryo development, ultimately leading to subfertility and even infertility. In the past decade, metabolism of mammalian oocytes during *in vitro* maturation has been extensively studied. For instance, Han et al. (9) indicated that melatonin supplementation significantly ameliorates the quality of maternal obesity-induced poor oocytes by reducing excessive ROS and meiotic errors in oocytes from high-fat diet (HFD) mice. Moreover, exogenous supplementation of nicotinamide mononucleotide (NMN) is a possible approach to protect oocytes from advanced maternal age-related deterioration (10). Despite significant research effort focusing on the effects of extrinsic nutrients, the intrinsic control of oogenesis by intracellular metabolites and metabolic enzymes has received little attention (11).

Herein, we obtain a dynamic UPLC/MS-based metabolome profile of porcine oocytes upon meiotic maturation. In parallel, transcriptomic profiling was employed to bolster the metabolomic data, corporately depicting the characterization of metabolic patterns in porcine oocytes during maturation. The current study not only provides a comprehensive multiple omics data resource, but also makes a theoretical foundation for discovery of biomarkers in the prediction of oocyte quality.

## 2 Materials and methods

### 2.1 Ethics statement

All experiments were approved by the Animal Care and Use Committee of Nanjing Agriculture University and were performed in accordance with Animal Research Institute Committee guidelines.

### 2.2 Oocyte collection and culture

Porcine ovaries were collected from prepubertal gilts at a local slaughterhouse and transported to the laboratory within 2h in prewarmed physiologic saline supplemented with 800 IU/mL gentamicin. The contents of antral follicles (3–5 mm in diameter) were aspirated with 20-gauge needles. Only fully grown oocytes with an evenly granulated cytoplasm, surrounded by at least three uniform layers of compact cumulus cells, were selected for experiments. The pooled column oocyte complexes (COCs) were washed three times with maturation medium, which was a TCM199 based medium supplemented with 10% porcine follicular fluid (PFF), 10 ng/ml of epidermal growth factor (EGF), 10 IU/ml of PMSG, 10 IU/ml of HCG, 0.57 mM cysteine, 0.91 mM sodium pyruvate, 3.05 mM D-glucose, 75 mg/ml of penicillin and 50 mg/ml of streptomycin. A group of COCs were transferred to 4-well plates containing 500 μL of maturation medium covered with 200 μL mineral oil, and incubated at 38.5°C under 5% CO_2_ in 95% humidified air for *in vitro* maturation (IVM).

After a period of incubation, COCs were removed in medium containing 0.02% (w/v) hyaluronidase at 38.5°C for 5 min. The denuded oocytes were isolated from COCs by repeatedly pipetting and then collected for subsequent experiment after 3~4 rinses.

### 2.3 Metabolomics

Respectively *in vitro* mature for 0h, 22h, 46h, denuded oocytes isolated from COCs after 3~4 rinses by Dulbecco’s Phosphate Buffered Saline (DPBS). Samples (400 oocytes per sample, 5 replicates for each stage) were transferred to the bottom of Eppendorf tubes, rapidly frozen by liquid nitrogen and stored at −80°C. In order to extract metabolite, samples were thoroughly mixed with 200 μL of methanol/water (80/20, vol/vol) and vortexmixed, followed by homogenized using a mechanical homogenizer. After incubation on ice for 10 min, samples were centrifugated at 16,000g for 15 min at 4°C. Supernatant was transferred to concentrated and desiccated, prior to storage at -80°C until instrumental analysis.

Mass spectrometry analysis was performed using the UltiMate3000 high -performance liquid chromatography system coupled with the Q-Exactive mass spectrometer. Subsequent chromatographic separation was performed on a Hypersil GOLD C18 column (1.9 μm, 100 mm ×2.1 mm), with temperature at 40°C. The binary solvent system used was solvent A of acetonitrile containing 0.1% formic acid, and solvent B of water containing 0.1% formic acid, which was at a flow rate of 0.4 mL/min. A multistep gradient was used for the metabolites analysis. Briefly, the column mobile phase was held 1% mobile phase A for 3 min (t=3 min), followed by an increase to 99% in 7 min lasting for 5 min (t=15 min), and finally immediately reduced to 1% and held for 2 min (t=17 min).

MS data were collected by the high-resolution mass spectrometer equipped with an electrospray ionization source. The instrument was operated in both positive and negative electrospray ionization (ESI) modes in a mass range 70-1050 m/z with a resolution of 70,000 for MS detection. The metabolites were identified by the comparison of accurate mass and retention time with commercial standard compounds using the author-constructed library. All samples were analyzed in a randomized fashion to avert complications of the injection order. ‘‘R’’ (V2.15) was used to perform all statistical analysis. The Orthogonal Partial Least Squares Discriminant Analysis (OPLS-DA) was conducted by SIMCA-P software (V14.0; Umetrics AB, Umea, Sweden) to observe Intra-group repeatability and between-group difference. T test (p < 0.05) coupled with a variable importance in projection (VIP) analysis (VIP >1.00) were considered statistically significant. KEGG Mapper (V4.1) (https://www.genome.jp/kegg/) was used to identified metabolic pathway information.

### 2.4 Transcriptomics

COCs that were collected at 0h, 22h, 46h of IVM were stripped of cumulus cells in the same manner. The single-cell samples (20 oocytes per sample, 4 replicates for each stage) were removed in tubes with lysis buffer containing protease inhibitor and ribonuclease inhibitor. Then we carried out the amplification by the SMART-Seq2 method.

For cDNA synthesis, an Oligo-dT primer was used to the reverse transcription reaction, followed by PCR amplification to enrich the cDNA and magbeads purification step to clean up the production. Primer sequences used include: oligo-dTV: 5’-AAGCAGTGGTATCAACGCAGAGTACTTTTTTTTTTTTTTTTTTTTTTTTTTTTTTVN-3’; Template Switching Oligo (TSO): 5’-AAGCAGTGGTATCAACGCAGAGTACATrGrG+G-3’; ISPCR: 5’-AAGCAGTGGTATCAACGCAGAGT-3’. Then the cDNA concentration was preliminarily measured using Qubit^®^ 3.0 Flurometer and Agilent 2100 Bioanalyzer to ensure the expected production with length around 1~2kbp. For library preparation, the pooled and purified cDNA was fragmented by sonication and then converted to sequencing libraries according to the standard Illumina library preparation protocol, including DNA fragmentation, end repair, 3’ ends A-tailing, adapter ligation, PCR amplification and library validation. PCR primers included P5 PCR Primer (5’TGATACGGCGAOCACCGAG) and P7 PCR Primer (5’AAGCAGAAGACGGCATACGAG). Library preparation integrity was verified with PerkinElmer LabChip^®^ GX Touch and Step OnePlus™ Real-Time PCR System. After libraries were qualified, sequencing libraries were performed on the Illumina Hiseq platform for PE150 sequencing.

Raw reads were first subjected to quality control with FastQC (v0.11.9). Based on the evaluation of the raw data, Trim galore (v0.6.4) was then used to trim sequencing adapters and filter out low-quality reads. The clean data were next aligned to the swine reference genome (Sscrofa11) by STAR (v2.7.6a) (12), followed by gene expression quantification by HTSeq (v1.99.2) (13) for counts and RSEM (v1.3.3) (14) for FPKM values. Differential expression analysis was performed with count data using the DESeq2 (v1.30.1) (15) R package, and the genes with absolute fold change > 1.5 and adjusted P value < 0.05 were treated as differentially expressed genes (DEGs). Enrichment analysis of DEGs for Gene Ontology (GO) terms and Kyoto Encyclopedia of Genes and Genomes (KEGG) pathways was conducted by DAVID (v2022q1) (16), and significantly enriched GO terms and KEGG pathways were satisfied with p-value < 0.05. FPKM values were used to generate heatmaps in R (v4.0.4). For network reconstruction between genes and metabolites, we only considered DEGs and differentially expressed metabolites (DEMs) for the network reconstruction. In brief, we took the intersection of top 300 DEGs between any paired time points, and, similarly, the intersection of DEMs for correlation analysis. We kept the connection between genes and metabolites if the Pearson’s correlation coefficients were greater than 0.95. Finally, the network between genes and metabolites based on correlation analysis was visualized with Cytoscape (v3.7.1).

### 2.5 Statistical analysis

The profiling of statistics was performed by the software GraphPad Prism (V7.0) for Windows. Student’s t test was used to identify the differential metabolites and genes, and data are presented as means ± SD (standard deviation), unless otherwise stated. A p value less than 0.05 was considered statistically significant.

## 3 Results

### 3.1 Metabolomic and transcriptomic profiling of porcine oocyte maturation

**Figure 1:**
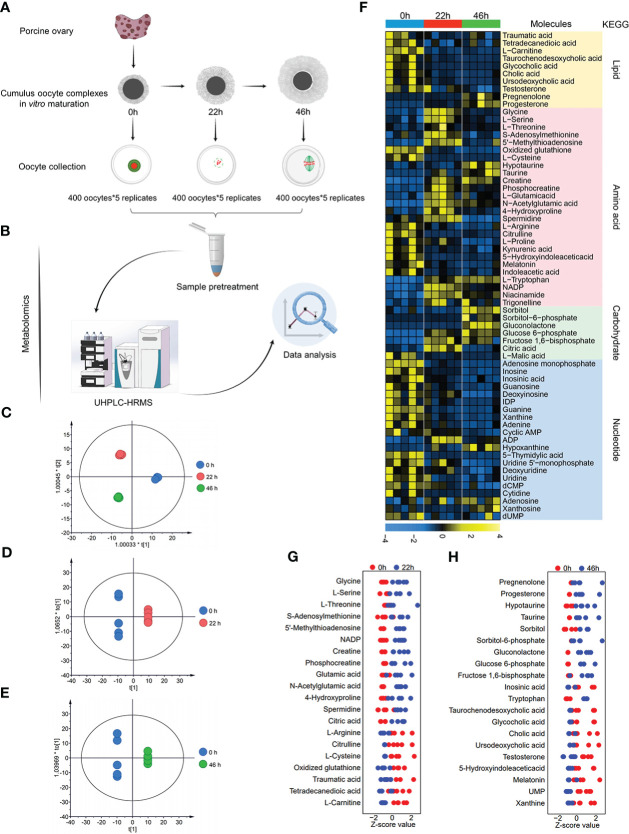
Metabolomic profiling of porcine oocyte maturation. **(A)** Collection of porcine oocytes cultured *in vitro* at key time points (0h, 22h, 46h). **(B)** Workflow for UPLC/MS-based metabolome profiling on porcine oocytes. **(C–E)** : OPLS-DA score plot for metabolomic datasets clearly distinguished oocyte samples from three time points. **(F)** Heatmap visualizing relative abundance of differential metabolites during porcine oocyte maturation, classified by metabolic pathway. **(G)** Z-score plots of 20 representative differential metabolites in oocytes *in vitro* cultured for 0h and 22h (meiotic resumption). **(H)** Z-score plots of 20 representative differential metabolites in oocytes *in vitro* cultured for 0 and 46h (meiotic maturation). Each color represents one phase, and each point represents one metabolite in one sample. The complete metabolomic data are available in **Supplementary Table 1**.

Although existing studies have identified several cellular pathways, we believe that the integrated profiling of metabolomics and transcriptomics contributes to a comprehensive insight of the metabolic dynamics as the oocytes progress through meiosis. Our previous study (17) has analyzed statistically the time-dependent nuclear maturation proportion of porcine oocyte during *in vitro* culture. About 96.3%, 89.2% and 73.2% of oocytes were at GV (0h), Pre-MI (24h), and MII (44h) stages, respectively. In the present study, we collected a great number of oocytes (totally 8,000 oocytes) isolated from cumulus-oocyte complexes (COCs) at three key time points (0h, 22h, 46h, **Figure 1A**). They were subjected to identification of intracellular metabolome using ultra-high-performance liquid chromatography-tandem high-resolution mass spectrometry (UHPLC-HRMS) (**Figure 1B**). A total of 441 metabolite components were detected, among which 197 differential metabolites were determined by the combination of a *t* test (p <0.05) and a variable importance in projection (VIP) analysis (VIP >1.00) (**Supplementary Table 1**). Scatted plots of orthogonal partial least-squares-discriminant analysis (OPLS-DA) clearly distinguished three groups, suggesting stage-dependent separation (**Figures 1C–E**). A heatmap was utilized to analyze and visualize the changes in metabolite level during meiotic maturation (**Figure 1F**). In order to extract significantly altered metabolic pathways, we mapped differentially expressed metabolites to Kyoto Encyclopedia of Genes and Genomes (KEGG), displaying the distinct metabolic patterns upon oocyte maturation. Generally, the levels of most amino acids and carbohydrate increased during meiotic maturation, whereas a notable reduction was observed in both lipid metabolites and nucleotides in the transition from GV to MII stage (**Figures 1F–H**).

**Figure 2:**
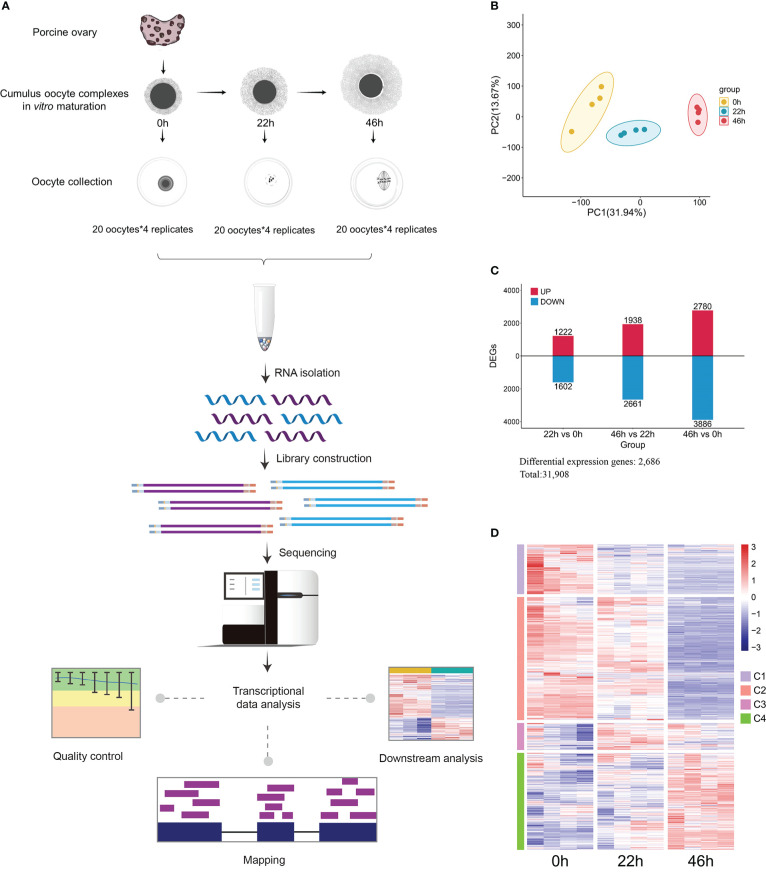
Transcriptomic profiling of porcine oocyte maturation. **(A)** Schematic overview of the workflow for transcriptome profiling in oocytes. **(B)** PCA plot for transcriptomic datasets separating 0h, 22h, and 46h oocyte samples. **(C)** Bar chart showing the up-regulated and down-regulated DEGs. **(D)** Heat map of hierarchical clustering of 2,686 differentially expressed genes from porcine oocytes cultured *in vitro* at key time points.

Gene transcription plays a crucial role in predicting and evaluating the dynamics of cellular processes during oocyte development. To explore the expression of the enzymes related to metabolism, we simultaneously conducted transcriptional profiling of oocytes at three stages mentioned above (**Figure 2A**). Principal Component Analysis (PCA) clearly showed stage-dependent separation of the three oocyte groups (**Figure 2B**). Changes in gene transcript abundance were assessed by differential expression analysis, and of the 31,908 genes identified, 2,686 exhibited significant alterations, based on p<0.05 couple with |log2(foldchange)| >0.59 (**Figures 2C, D** and **Supplementary Table 2**). Gene Ontology (GO) and KEGG pathway enrichment analyses were conducted for differential expression genes, exhibiting a significant enrichment for numerous gene categories critical for metabolic pathways (**Supplementary Figures 1A, B**). Differentially expressed genes were mapped to KEGG metabolic pathways, and 9 significantly altered pathways were uncovered, involving metabolism of bile acid, tryptophan, purine and pyrimidine, and etc. (**Supplementary Figures 1C–I**). Collectively, by integrating metabolomics and transcriptomics, we have provided a broad picture of global metabolic characteristics and dynamic changes in different pathways during porcine oocyte maturation.

### 3.2 Lipid metabolism during oocyte maturation

**Figure 3:**
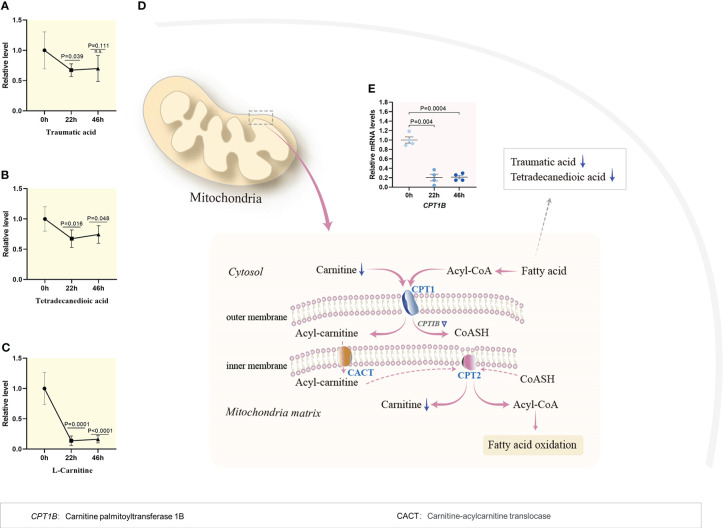
Reduced fatty acid beta oxidation during meiotic resumption. **(A–C)** Relative levels of metabolites related to fatty acid oxidation in oocytes at three time points. **(D)** Schematic diagram of carnitine shuttle system and utilization of fatty acid during porcine oocyte maturation, derived from metabolomics and transcriptomics. Metabolites decreased in oocytes during meiotic resumption are indicated by bold blue arrows. Differential expression genes decreased are indicated by blue triangles **(E)** Dynamic changes in the relative level of *CPTIB* during meiotic maturation. Error bars, SD. Student’s t test was used for statistical analysis in all panels, comparing to GV. n.s., not significant.

The dynamic characteristics of lipid metabolism during porcine oocyte maturation were poorly defined. As shown in **Figure 1F**, temporal metabolome profiles clearly displayed the alternations in level of lipid metabolites as the oocytes progress through meiosis, with enrichment primarily in fatty acid beta-oxidation (**Figure 3**) and metabolism of bile acid (**Supplementary Figure 2**) and steroid hormones (**Supplementary Figure 3**). The content of lipid metabolites involved in fatty acid beta-oxidation (i.e., traumatic acid, tetradecanedioic acid, carnitine, **Figures 3A–C**) declined dramatically during meiotic resumption, whereas the abundance of bile acid and steroid hormones metabolism-related products (i.e., cholic acid, glycocholic acid, ursodeoxychoic acid, testosterone, and progesterone, **Supplementary Figures 2** and **3**) displayed a notable reduction in MII oocytes compared to GV oocytes. Such the dynamic change of lipid-related products might play a vital role in nutrient adjustment, hormone regulation, and cellular homeostasis (18).

#### 3.2.1 Reduced fatty acid beta oxidation during meiotic resumption

Fatty acids are indispensable as substrates for energy production and the synthesis of most lipids. Once inside the outer mitochondrial membrane, the long chain acyl-CoA combines with carnitine and converts to acylcarnitine, which is catalyzed by carnitine palmitoyl transferase 1 (CPT1). Through a carnitine-acylcarnitine translocase (CACT), acylcarnitine couriers across the inner mitochondrial membrane, where carnitine is removed by carnitine palmitoyl transferase II (CPT2), and the acyl-CoAs enters the fatty acid oxidation pathway to produce ATP. Of note, metabolomic profiling revealed that the content of fatty acids declined during meiotic resumption (**Figures 3A, B**), and simultaneously, the carnitine level experienced a significant decline by 83% (**Figure 3C**). Consistent with this observation, the mRNA level of carnitine palmitoyltransferase 1, markedly decreased during meiotic resumption based on transcriptomic data (**Figures 3D, E**). Du et al. showed that removal of lipid droplets from porcine oocytes does not significantly affect the developmental rates of blastocysts (19). Collectively, these findings indicate that the activity of fatty acid β-oxidation pathway is suppressed during porcine oocyte maturation.

#### 3.2.2 Diminished activity of Bile Acid Biosynthesis during Maturation

In mammals, the products of cholesterol metabolism mainly are bile acids, steroid hormones and their derivatives (20–22). Bile acids are increasingly being appreciated as complex metabolic integrators and signaling factors (23). Interestingly, our metabolomic analysis revealed that levels of metabolites involved in bile acid metabolism exhibited the dramatic changes during porcine oocyte maturation. In specific, the abundance of cholic acid, glycocholic acid, ursodeoxychoic acid, and taurochenodeoxycholic acid was subjected to decrease by around 90% in matured oocytes as compared to GV oocytes (**Supplementary Figures 2A–E**). Moreover, the mRNA level of *HSD17B4*, which encoded 17β-hydroxysteroid dehydrogenase type 4 in the primary bile acid biosynthesis pathway, also experienced the significant downregulation accompanying with oocyte maturation (**Supplementary Figure 2F**), indicative of the diminished activity of bile acid biosynthesis.

#### 3.2.3 Elevated levels of steroid hormones in meiotic porcine oocytes

Pregnenolone, as the common steroidal precursor molecule, mediates a wide variety of vital developmental and physiological functions. Accumulated evidence has suggested that cumulus cells, stimulated by follicle-stimulating hormone (FSH), luteinizing hormone (LH), or both, give rise to steroid hormones, especially progesterone, which is integral to meiotic maturation of porcine oocytes (24, 25). Here, we found that the abundance of pregnenolone and progesterone underwent a remarkable increase (pregnenolone: ~25-fold; progesterone: ~40-fold) in maturing oocytes (**Supplementary Figures 3A, B**). Interestingly, the metabolomic data showed the significantly decreased levels of testosterone in MII oocytes (**Supplementary Figure 3C**). Of note, the mRNA levels of *HSD17B2, HSD17B3, HSD11B1L* and *STS* were all decreased as the oocytes enter meiosis (**Supplementary Figures 3D–H**), indicating that increased abundance of pregnenolone and progesterone may be due to the downregulation of related pathways in oocyte during maturation. Altogether, the results imply that the elevated pregnenolone and progesterone might play essential roles in controlling meiotic maturation of porcine oocytes.

### 3.3 Diverse metabolic characteristics of amino acid during oocyte maturation

**Figure 4:**
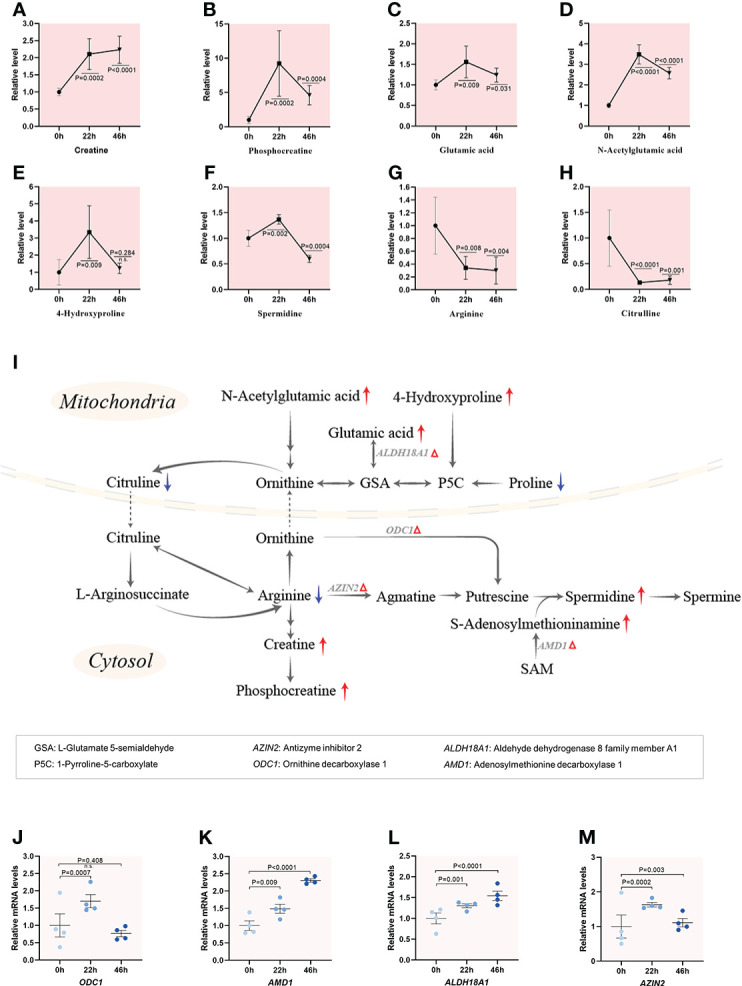
Enhanced catabolism of arginine and proline during meiotic resumption.**(A–H)** Relative levels of metabolites involved in arginine and proline metabolism in oocytes at three time points. **(I)** Schematic diagram of arginine and proline metabolism during meiotic resumption, based on metabolomics and transcriptomics. The red and blue arrows denote the metabolites that were upregulated and downregulated, respectively. Differential expression genes increased are indicated by red triangles. **(J–M)** Relative levels of differential expression genes related to arginine and proline metabolism during meiotic resumption. Error bars, SD. Student’s t test was used for statistical analysis in all panels, comparing to GV. n.s., not significant.

**Figure 5:**
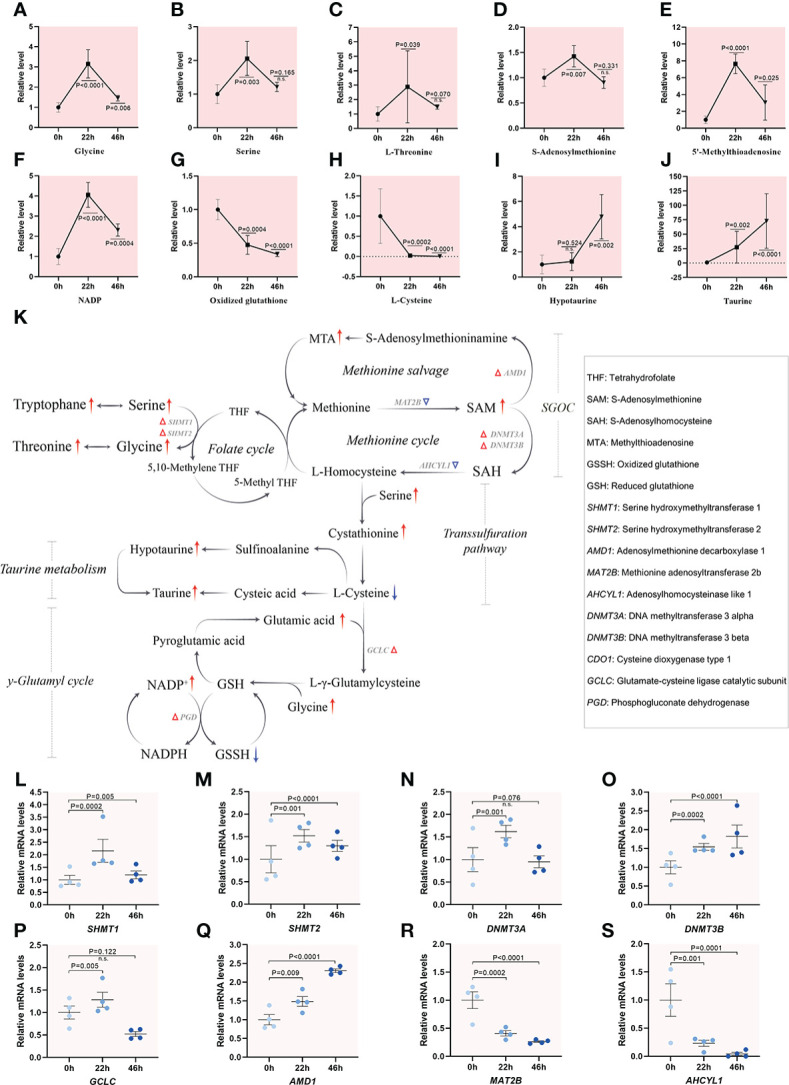
Active one-carbon metabolism in porcine oocytes. **(A–J)** Relative levels of metabolites related to one-carbon metabolism in oocytes at three time points. **(K)** Overview of the metabolic processes of one-carbon metabolism in porcine oocytes upon meiotic resumption, derived from metabolomics and transcriptomics. The red and blue arrows denote the metabolites that are upregulated and downregulated, respectively. Differential expression genes increased and decreased are indicated by red and blue triangles, respectively. **(L–S)** Relative levels of representative genes involved in inputs and outputs of one-carbon metabolism during meiotic resumption. Error bars, SD. Student’s t test was used for statistical analysis in all panels, comparing to GV. n.s., not significant.

**Figure 6:**
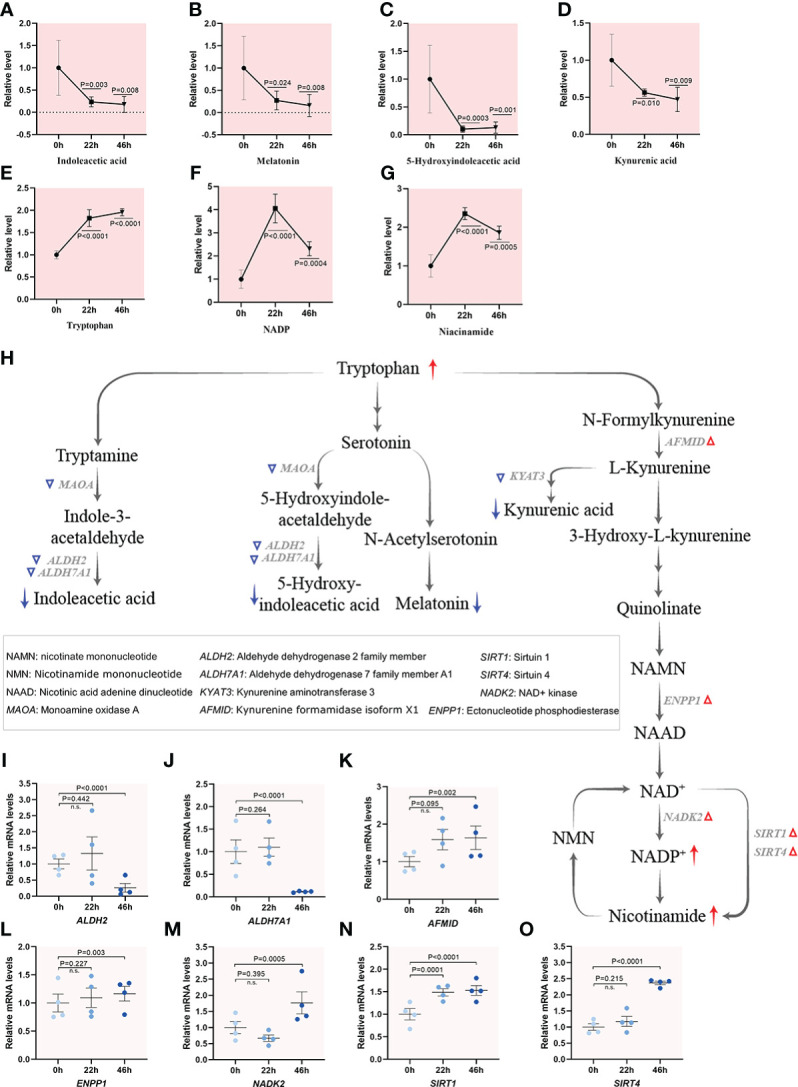
Tryptophan utilization during porcine oocyte maturation. **(A–G)** Relative levels of metabolites related to tryptophan metabolism in oocytes at three time points. **(H)** Schematic diagram of tryptophan metabolism during meiotic maturation, derived from metabolomics and transcriptomics. The red and blue arrows denote the metabolites that are upregulated and downregulated, respectively. DEGs increased and decreased are indicated by red and blue triangles, respectively. **(I–O)** Relative levels of representative differential expression genes involved in tryptophan utilization for maturation of porcine oocytes. Error bars, SD. Student’s t test was used for statistical analysis in all panels, comparing to GV. n.s., not significant.

Amino acids play important roles in the metabolism of all organisms, including protein synthesis, energy production (26), organic osmolytes (27), and intracellular buffer (28). Nevertheless, very little is known about metabolic dynamics of amino acids during porcine oocyte maturation. A combined metabolome and transcriptome analysis of porcine oocyte reveals the diverse characteristics of amino acid metabolism during maturation (**Figures 4**–**6**; **Supplementary Figure 4**), reflecting the sophisticated and fine-tuned regulation in oocytes. Four metabolic pathways significantly changed in meiotic oocytes were presented below.

#### 3.3.1 Enhanced catabolism of arginine and proline during meiotic resumption

Proline can be converted to citrulline in mitochondria, and then citrulline is transported to the cytosol, where serves as a precursor for synthesis of arginine. Arginine can be further metabolized to produce creatine and polyamines, thus constituting a quantitatively minor pathway for polyamine synthesis in mammals (29). Temporal metabolome profiles exhibited that 6 (i.e., creatine, phosphocreatine, glutamic acid, acetylglutamic acid, 4-hydroxyproline, spermidine) out of 8 key components of arginine and proline metabolism we detected showed the significant accumulation upon meiotic resumption (**Figures 4A–F**). In striking contrast, the levels of both arginine and proline were drastically decreased when oocyte enter meiosis (**Figures 4G, H**). Furthermore, the expression of those differential expression genes (*ODC1*, *AMD1*, *ALDH18A1* and *AZIN2*) responsible for arginine/proline metabolism were all upregulated during oocyte maturation (**Figures 4I–M**). Such a metabolic flux strongly indicates the enhanced catabolism of arginine and proline during porcine oocyte meiosis. A series of findings have demonstrated that metabolism of arginine and proline plays important roles in cellular signaling regulation (30), synthesis of polyamines (31), as well as antioxidative reactions (32).

#### 3.3.2 Active one-carbon metabolism in porcine oocytes

Serine donates the carbon atom to tetrahydrofolate (THF), initiated by serine hydroxymethyltransferase (SHMT), forming glycine and 5,10-methylene-THF, which starts the folate cycle. Methyl groups derived from one-carbon metabolism are transmitted into homocysteine to perform methionine cycle (33). Metabolomic analysis revealed that the levels of metabolites that participate in one-carbon metabolism were all elevated upon meiotic resumption, and then reduced in maturing oocytes (**Figures 5A–E**). For instance, 8-fold and 1.5-fold increase in methylthioadenosine (MTA) involved in methionine cycle, and S-adenosylmethionine (SAM), a methyl group donor in many biochemical reactions, were observed, respectively. The transsulfuration pathway results in the transfer of the sulfur atom from methionine to serine to synthesize cysteine, a key constituent of the powerful antioxidant molecule glutathione (GSH) synthesis (2) and taurine metabolism. In line with this notion, we observed the altered content of NADP and oxidized glutathione during meiotic resumption (**Figures 5F–H**). The alternations in γ-glutamyl cycle could, at least in part, be attributed to maintaining the appropriate NADPH/NADP^+^ ratio as well as cellular redox homeostasis (34). In addition, cysteine dioxygenase 1 (CDO1) converts cysteine to sulfinoalanine, which can be further converted to hypotaurine for taurine production (35). A progressive increase was observed for both hypotaurine (~5-fold) and taurine (~75-fold) during maturation, implying active taurine metabolism (**Figures 5I, J**). Correspondingly, transcriptome profiling data also identified a ~2-fold increase in 6 out of 8 genes related with the pathways mentioned above during meiotic resumption (**Figures 5K–S**). One-carbon metabolism encompasses a complicated metabolic network and generates diverse outputs that can be subdivided into different specific systems, including the methionine salvage, methionine cycle, transsulfuration pathway, γ-glutamyl cycle and taurine metabolism. The output metabolites of one-carbon metabolism participate in synthesis of proteins, purine and pyrimidine nucleotides, phospholipids, as well as in the control of cellular epigenetic landscape *via* DNA and histone methylation (36). Collectively, integrated analysis of metabolomic and transcriptomic profiling reveals the active one-carbon metabolism during meiotic resumption in porcine oocytes.

#### 3.3.3 Tryptophan utilization during meiotic maturation

Beyond its role in as a building block of proteins, tryptophan undergoes two metabolic routes: retaining the indole ring or breaking the indole ring to form kynurenine, giving rise to a complex metabolic pathways (37). Interestingly, all bioactive indole compounds displayed a notable decrease during oocyte maturation. For instance, the levels of indoleacetic acid, melatonin and 5-hydroxy indoleacetic acid decreased gradually as the oocytes progress through meiosis, suggesting an overall decrease in metabolic activity (**Figures 6A–C**). On the other hand, the majority of free tryptophan is metabolized along the kynurenine pathway, which is converted to quinolinic acid and nicotinamide adenine dinucleotide (NAD^+^) under normal conditions. The level of kynurenic acid, a product of kynurenine metabolism, also displayed reduction in mature oocytes (**Figure 6D**). Remarkably, ~4-fold and ~2-fold increases during oocyte maturation were observed for both NADP^+^, the phosphorylated form of NAD^+^ through NAD kinase (NADK), and nicotinamide that catalyzed by Sirtuins in NAD salvage pathway (**Figures 6E–G**). Consistent with these metabolic alternations, the mRNA levels of the enzymes responsible for biosynthesis of indole compounds were downregulated during oocyte maturation accordingly (**Figures 6H–J**). However, the mRNA levels of *NADK2*, *ENPP1*, *SIRT1* and *SIRT4* were elevated during meiotic maturation (**Figures 6K–O**), indicative of active NAD synthesis. Considerable evidence has accumulated that NAD^+^ and NADP^+^ may be among the fundamental common mediators of various biological processes, including energy metabolism, epigenetics, and disease states (38, 39). Altogether, integrated profilings highlighted the different flux of tryptophan during porcine oocyte maturation, revealing the suppressed biosynthesis of indole compounds and the enhanced NAD *de novo* synthesis pathway.

### 3.4 Enhancement of carbohydrate metabolism activity during oocyte maturation

**Figure 7:**
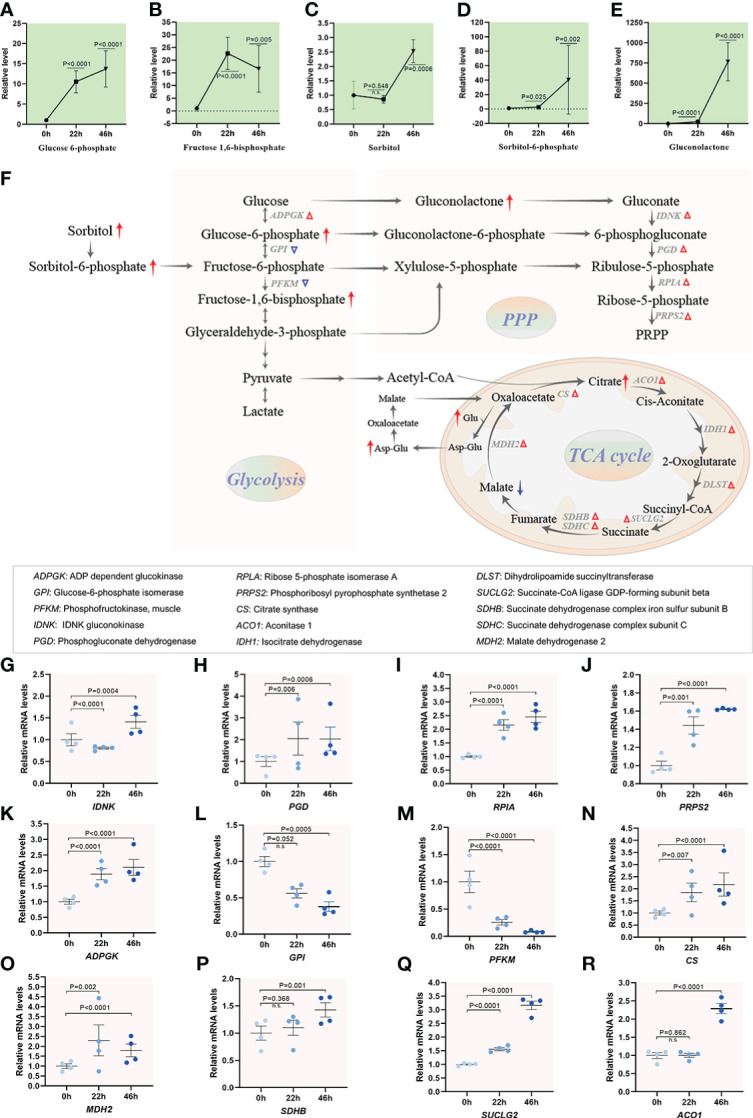
Carbohydrate metabolism activity during oocyte maturation. **(A-E)** Relative levels of metabolites related to carbohydrate metabolism in oocytes at three time points. **(F)** Schematic diagram of carbohydrate metabolism during meiotic maturation, derived from metabolomics and transcriptomics. The red and blue arrows denote the metabolites that are upregulated and downregulated, respectively. Differential expression genes increased and decreased are indicated by red and blue triangles. **(G–R)** Relative levels of differential expression genes involved in carbohydrate metabolism during oocyte maturation. Error bars, SD. Student’s t test was used for statistical analysis in all panels, comparing to GV. n.s., not significant.

In mammals, a close relationship between carbohydrate metabolism and maturation process in oocyte has been proposed (40). However, the metabolite dynamics of carbohydrate during oocyte maturation remain poorly studied. Glucose, utilized through both pentose phosphate pathway (PPP) and tricarboxylic acid (TCA) cycle, is essential for oogenesis in pigs (41, 42). Our metabolomic analysis demonstrated that several key components of glycolysis and PPP experienced dramatic alterations upon meiotic maturation. For instance, an ~15-fold-increase was observed for glucose 6-phosphate and fructose 1,6-bisphosphate, an ~40-fold-increase in sorbitol 6-phosphate, and particularly ~800-fold increase in gluconolactone were identified in MII oocytes compared to GV oocytes (**Figures 7A–E**). Meanwhile, transcriptome analysis identified the increased mRNA levels of 5 (i.e., *IDNK, PGD, RPIA, PRPS and ADPGK*) out of 7 enzymes related to the metabolic pathways mentioned above (**Figures 7F–M**). In addition, as a metabolism hub, TCA cycle serves to connect the processes of glycolysis, amino acid synthesis and other biosynthetic pathways (43). The level of malate was decreased in maturing oocytes ( **Supplementary Figure 5A**). Nonetheless, the abundance of citrate underwent ~3-fold-increase during meiotic resumption (**Supplementary Figure 5B**). Consistent with this observation, the abundance of differential expression genes (i.e., *CS, MDH2, SDHB, SUCLG2 and ACO1*) involved in TCA cycle showed the significant upregulation during meiotic maturation, implying the enhancement of TCA activity (**Figures 7N–R** and **Supplementary Figures 5C–E**). Carbohydrate metabolism occupies the core of bio-molecular metabolism, and the substrates involved in carbohydrate metabolism provide the essential saccharides and energy for oocyte maturation. Cumulatively, our results indicate the active carbohydrate metabolism, specifically, the PPP and TCA cycle, during porcine oocyte maturation.

### 3.5 A progressive decrease in nucleotide metabolism during oocyte maturation

**Figure 8:**
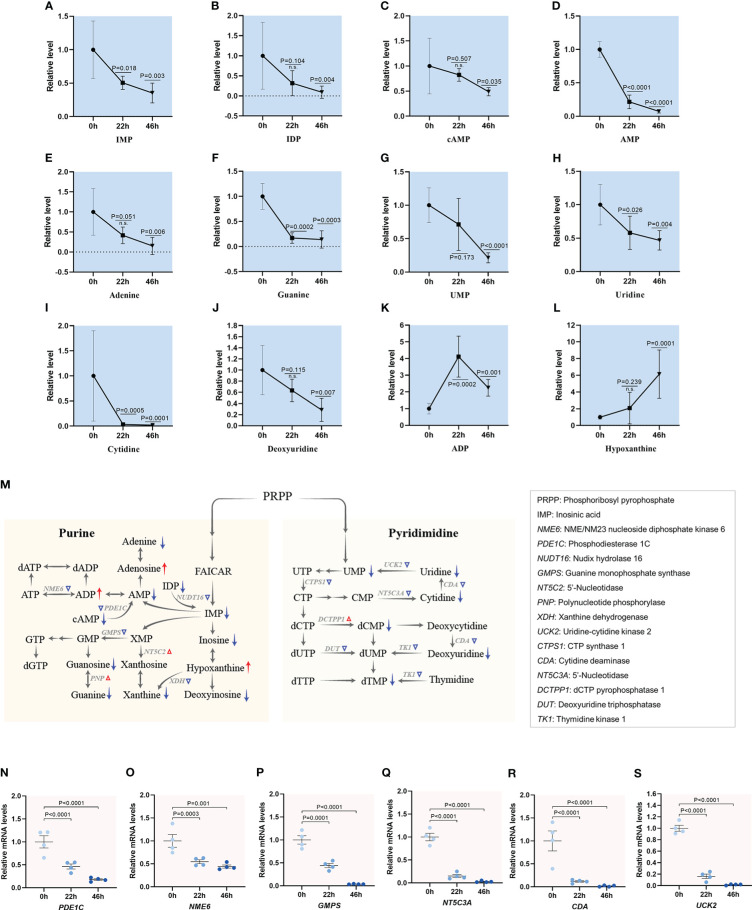
Metabolic changes in nucleotides during oocyte maturation. **(A-L)** Relative levels of metabolites related to nucleotide metabolism in oocytes at three time points. **(M)** Schematic diagram of nucleotide metabolism during meiotic maturation, derived from metabolomics and transcriptomics. The red and blue arrows denote the metabolites that are upregulated and downregulated, respectively. DEGs increased and decreased are indicated by red and blue triangles, respectively. **(N–S)** Relative levels of DEGs involved in glucose metabolism during oocyte maturation. Error bars, SD. Student’s t test was used for statistical analysis in all panels, comparing to GV. n.s., not significant.

Purine and pyrimidine nucleotides play critical roles as precursors for the synthesis of DNA and RNA, major energy carriers, and core elements of cofactors in metabolic pathways (44). In the *de novo* synthesis of purine nucleotides, activated ribose-5-phosphate (R5P) is then converted to Inosinic acid (IMP), which is further transformed into AMP and GMP. Similarly, the *de novo* synthesis of pyrimidine ends with the production of UMP, from which other pyrimidine nucleotides are converted. Temporal metabolome profiles clearly revealed that out of 17 differential metabolites involved in nucleotide metabolism, the abundance of 15 experienced the significantly decrease during meiotic maturation (**Figures 8A–L** and **Supplementary Figures 6A–E**). Consistent with the metabolite changes, the majority of enzymes responsible for purine and pyrimidine metabolism presented the reduced mRNA levels in matured oocytes (**Figures 8M** **–S** and **Supplementary Figures 6F–M**). For instance, transcriptomic data showed a 95% decrease in GMP synthase (GMPS), an enzyme in guanine ribonucleotide biosynthesis, and cytosolic 5’-nucleotidase 3A (NT5C3A), an enzyme in purine salvage pathway, in matured oocytes compared to GV oocytes. Together, the results clearly reveal the progressive reduction in nucleotide metabolism during porcine oocyte maturation.

## 4 Discussion

**Figure 9:**
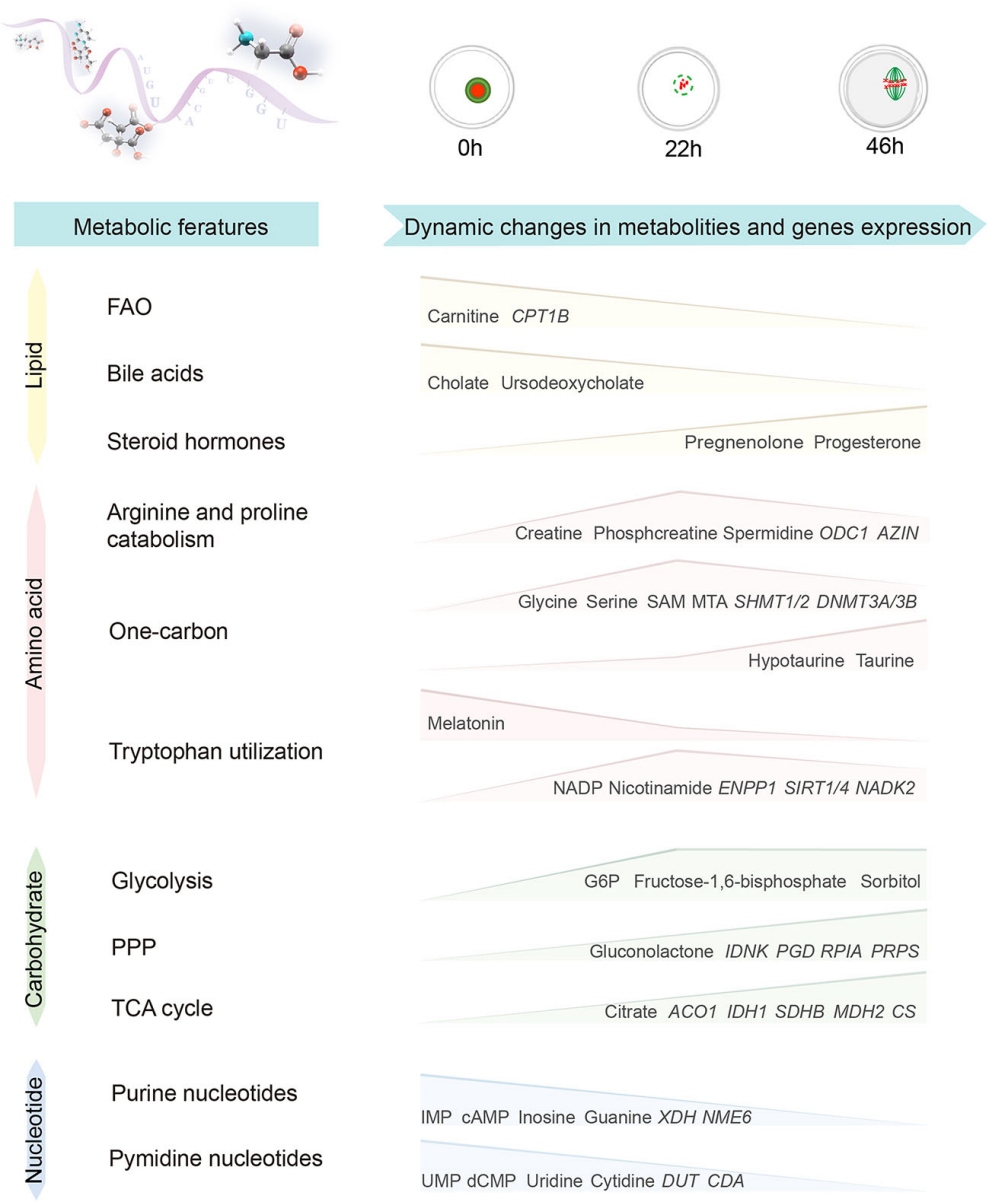
Characteristics of global metabolic patterns during porcine oocyte maturation. Diagram illustrating the dynamic changes in metabolites and gene expression during porcine oocyte maturation.

Metabolomics can reflect the state and events of metabolism within cells (11), however, profiling of metabolites in mammalian oocytes and embryos is still in the initial stage. Herein, we conducted an integrated analysis of metabolomics and transcriptomics by isolating porcine oocytes at key stages, illustrating the characteristics of global metabolic patterns in porcine oocytes. Remarkably, the most significantly altered pathways were identified during oocyte maturation (**Figure 9**), including (1) reduced fatty acid beta oxidation (2), diminished bile acid biosynthesis (3), enhanced catabolism of arginine and proline (4), active one-carbon metabolism (5), different flux of tryptophan utilization, and (6) a progressive decline in nucleotide metabolism. These dynamic changes in different pathways not only uncover the metabolic networks regulating oocyte development, but also are helpful for both prediction in biomarkers of oocyte quality and improvement of female fertility.

Enhanced *de novo* synthesis of NAD and active one-carbon metabolism strongly imply the ties between oocyte metabolism and epigenetic modifications. We discovered that the level of NMA, involved in active *de novo* synthesis of NAD, and Sirtuins, a family of NAD^+^-dependent histone deacetylases, experience a dramatically increase during maturation. Accumulating evidence suggests that the Sirtuins family is essential for modulation of histone acetylation status, thereby contributing to maintaining the meiotic apparatus in mammalian oocytes (45, 46). On the other hand, our study demonstrated the significant upregulation of one-carbon metabolism at both the metabolic and transcriptional levels. As cosubstrates of chromatin and DNA modifying enzymes, S-adenosylmethionine (SAM) serves as the high-energy methyl donor for requirement of methylation modification. There is now a growing appreciation that the dynamic change of metabolites influences the deposition and removal of epigenetic modifications. Obesity, diabetes, as well as many developmental failures and disorders, have been found to be associated with oocyte epigenetic abnormalities (47, 48). However, prevention and correction of these maternally transmitted nongenetic disorders remain challenging because of the lack of a comprehensive investigation of metabolite dynamics during oocyte maturation. Our finding provides a mechanistic framework for dissecting how oocyte metabolism could impact the complex, yet highly coordinated, epigenetic alterations during maturation. In the future, emphasis should be placed on genetic manipulations of key metabolic genes to determine how specific metabolites interact with epigenetic alternation in oocyte development.

Beyond its role as precursor for the synthesis of biological molecules, arginine plays a vital role in regulating many metabolic pathways that are vital to reproduction, growth, and health (49). In the current study, we found the enhanced catabolism of arginine and proline during oocyte maturation. Supplementation of diet with arginine during early gestation has been reported to improve implantation sites, embryonic survival, and litter size, indicating beneficial effects of optimal arginine and proline metabolism on reproductive performance (49, 50). Recently, Chen et al (51) discovered that oocytes in low reproductive performance (LRP) sows have reduced uptake and metabolism of arginine and proline, resulting in inhibition of oocyte development. It is conceivable that maternal environments change such as obesity and diabetes, may disrupt the metabolic patterns of amino acids, particularly uptake of arginine and proline, resulting in the impairment of oocyte quality and offspring development. Hence, the altered abundance of metabolites involved in catabolism of arginine and proline provides the potential interventional targets for the discovery of oocyte quality biomarkers.

Despite the findings in this study encouraging for further exploration, there are some potential limitations that should be acknowledged. Of particularly note is that we only describe the alternations of differential metabolites identified in the present study, non-differential metabolites such as melatonin, methionine and nicotinamide mononucleotide, among others, which may also be very necessary for porcine oocyte maturation. Moreover, it is difficult to decipher the alterations in individual metabolites, as they may participate in several pathways. For a given metabolite, elevation in its concentration could represent increased production, decreased consumption, or both (52). Thus, we have inferred changes during maturation in metabolic pathways based on the coordinated alterations in levels of metabolites and transcripts of genes encoding metabolic enzymes. In addition, we showed the changes in metabolism in multiple dimensions; however, metabolic activities are strongly influenced by allosteric regulation and posttranslational modifications in key enzymes, and these modifications often play an important role in the regulation of cell signal transduction, development, and other processes. The correlations of these dimensions were lacking because of the lack of a well-developed workflow for analysis ranging from genome to metabolome. Finally, somatic granulosa cells and cumulus cells surround oocytes, and their interactions are important for oogenesis (53). Metabolomic analysis will provide the next large set of clues to further our understanding of metabolic control of oocyte development.

## 5 Conclusion

In summary, we applied metabolomics and transcriptomics to uncover an integrated global picture of the metabolic characteristics during porcine oocyte maturation. Our dataset provides insights into the processes occurring in meiotic oocytes. We are optimistic that these findings will promote the development of approaches to manipulate the newly identified metabolic modules to improve oocyte quality and provide tremendous opportunities to define better germ cell culture systems.

## Data availability statement

The data presented in the study are deposited in the GEO repository, accession number GSE222399.

## Ethics statement

All experiments were approved by the Animal Care and Use Committee of Nanjing Agriculture University and were performed in accordance with Animal Research Institute Committee guidelines.

## Author contributions

MG, LG, and QW conceived the projects. MG and MC contributed to the metabolomics profiling; MG, QC, and XW contributed to the transcriptomics profiling. MG, SZ, HW, and WY assisted with the oocytes collection and *in vitro* culture experiments. MG and LG wrote and QW revised the manuscript. All authors contributed to the article and approved the submitted version.
