# Supplementary figures and images for: Integration of parallel metabolomics and transcriptomics reveals metabolic patterns in porcine oocytes during maturation
Source: Front Endocrinol (Lausanne). 2023 Feb 1;14:1131256. doi: 10.3389/fendo.2023.1131256 (PMC9929430; doi:10.3389/fendo.2023.1131256)

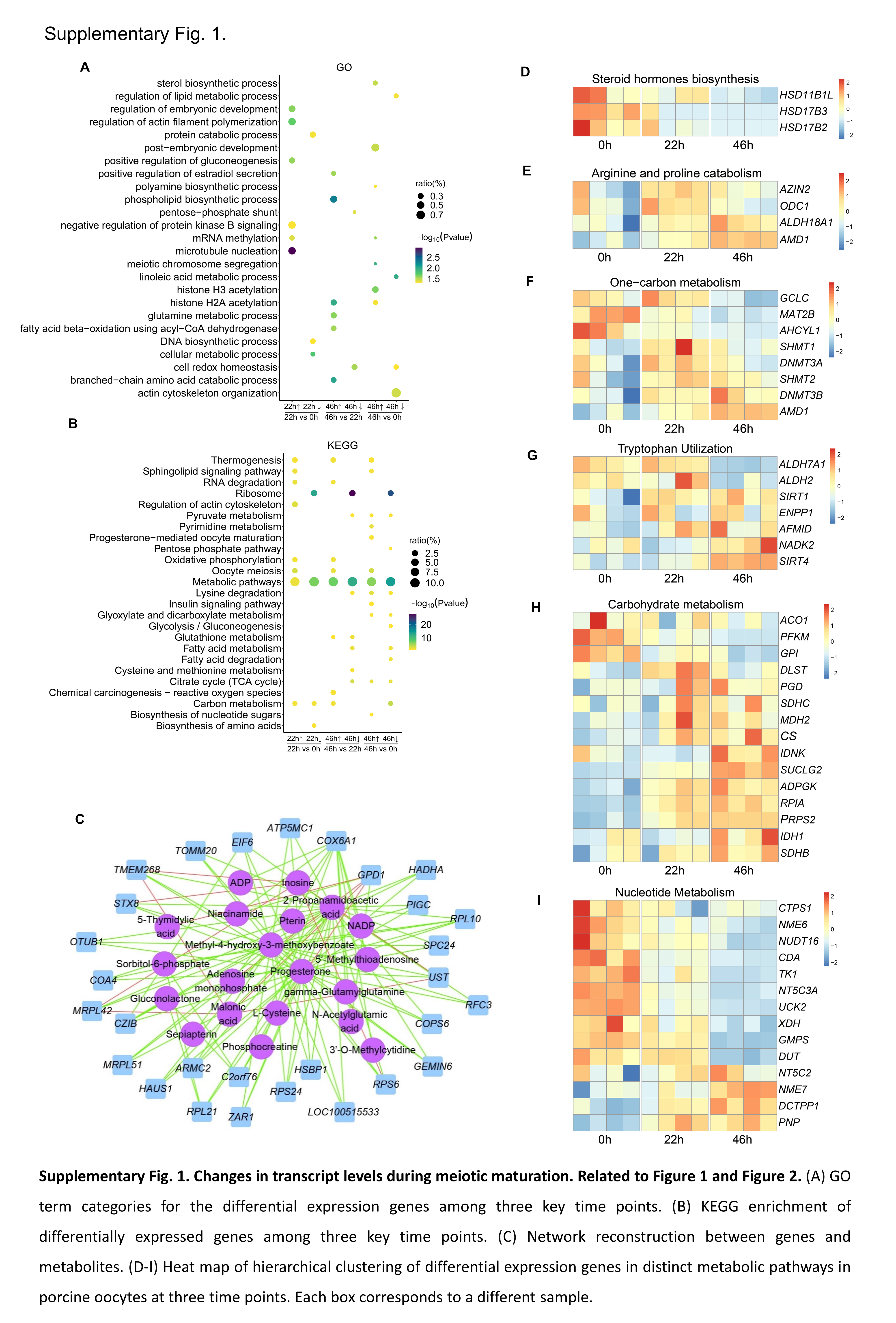

Supplement: Supplementary file 3 [file Image_1.tif]

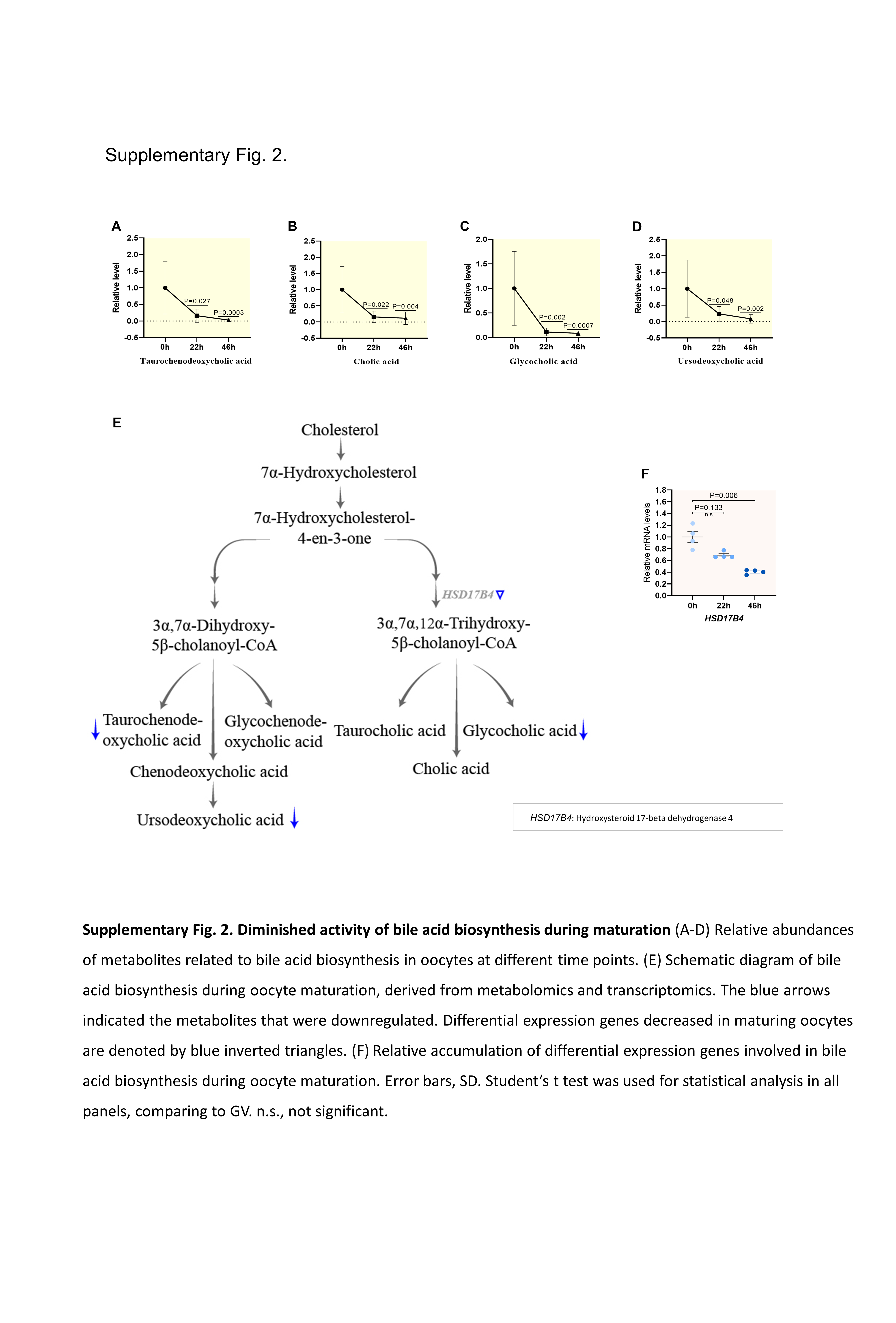

Supplement: Supplementary file 4 [file Image_2.tif]

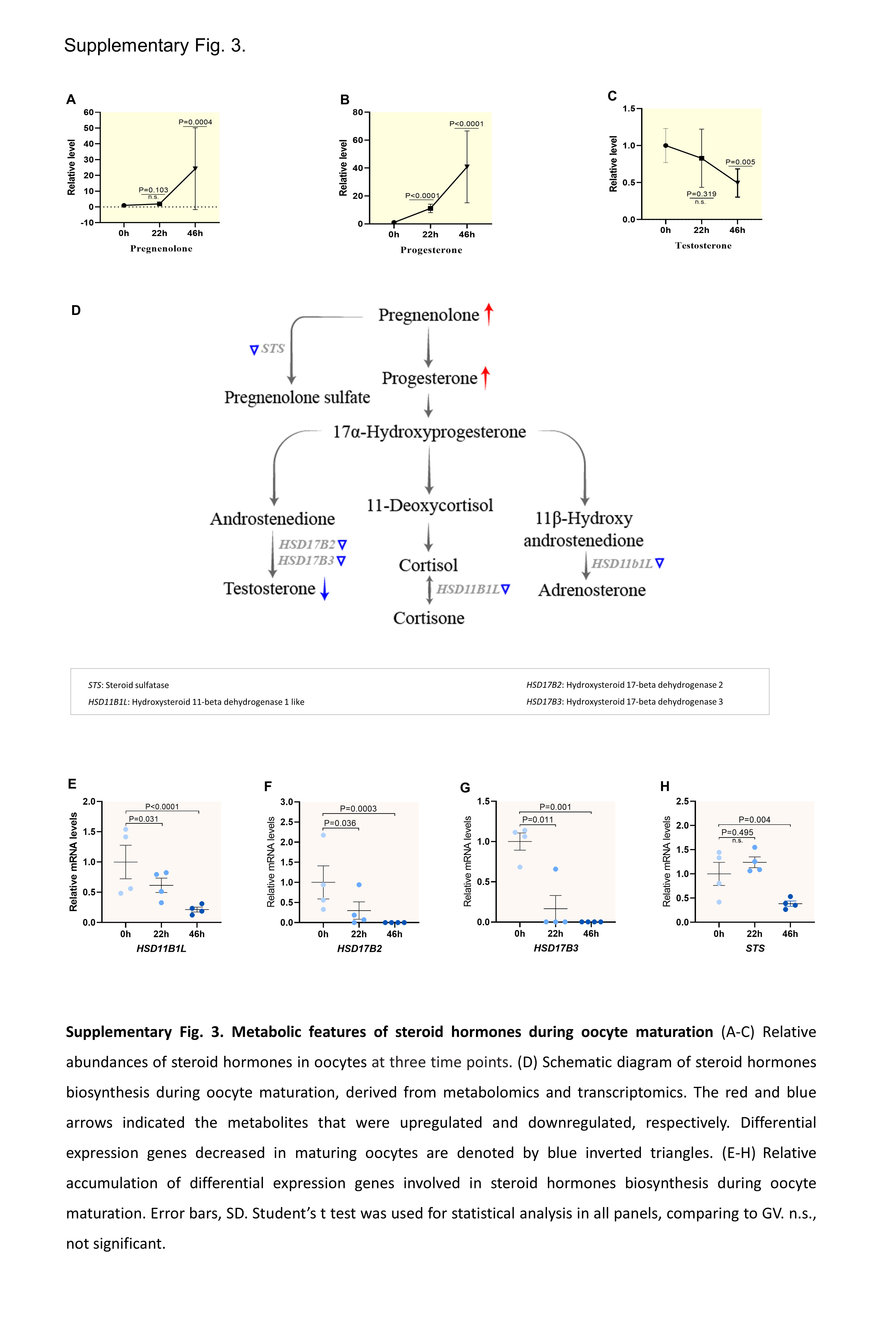

Supplement: Supplementary file 5 [file Image_3.tif]

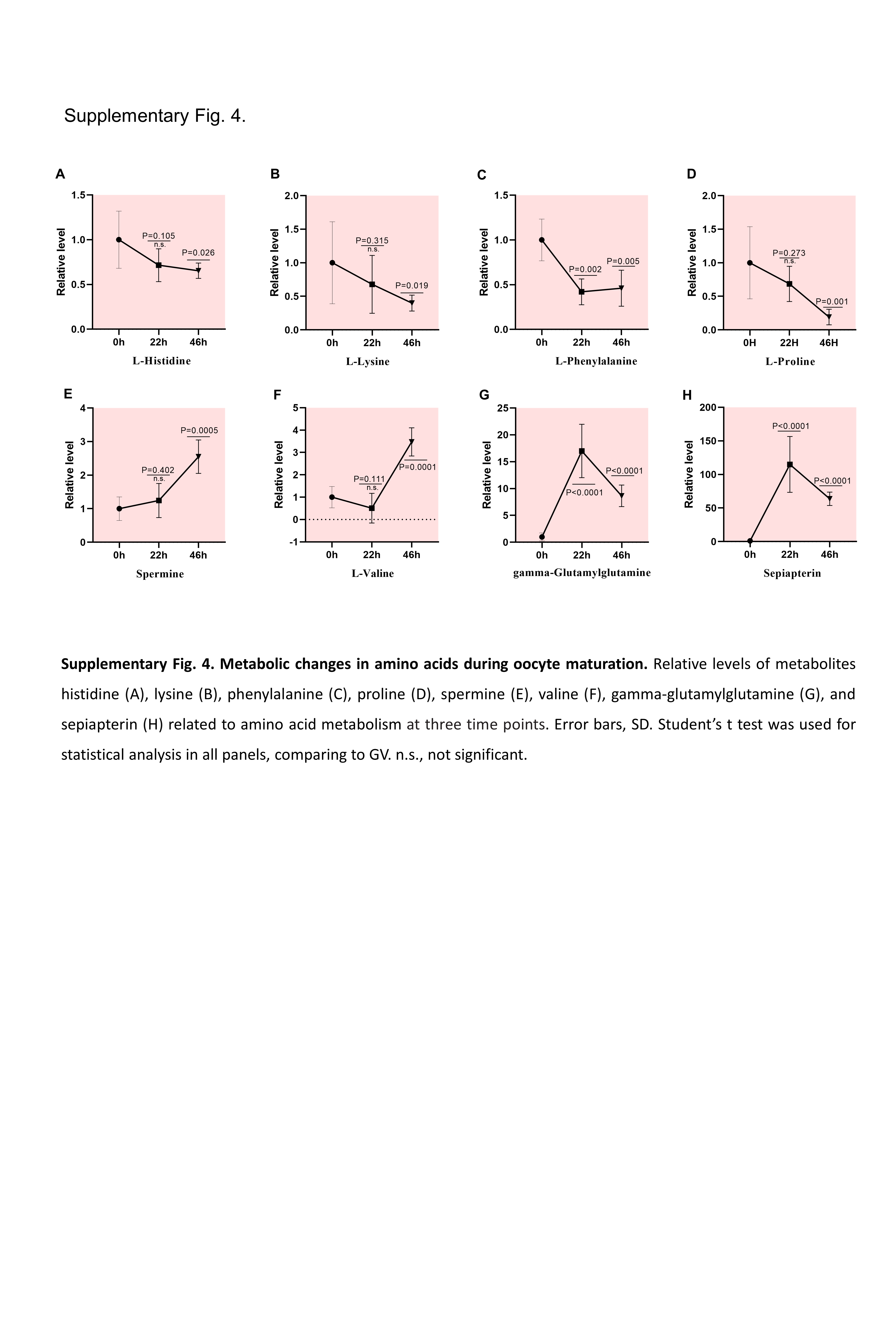

Supplement: Supplementary file 6 [file Image_4.tif]

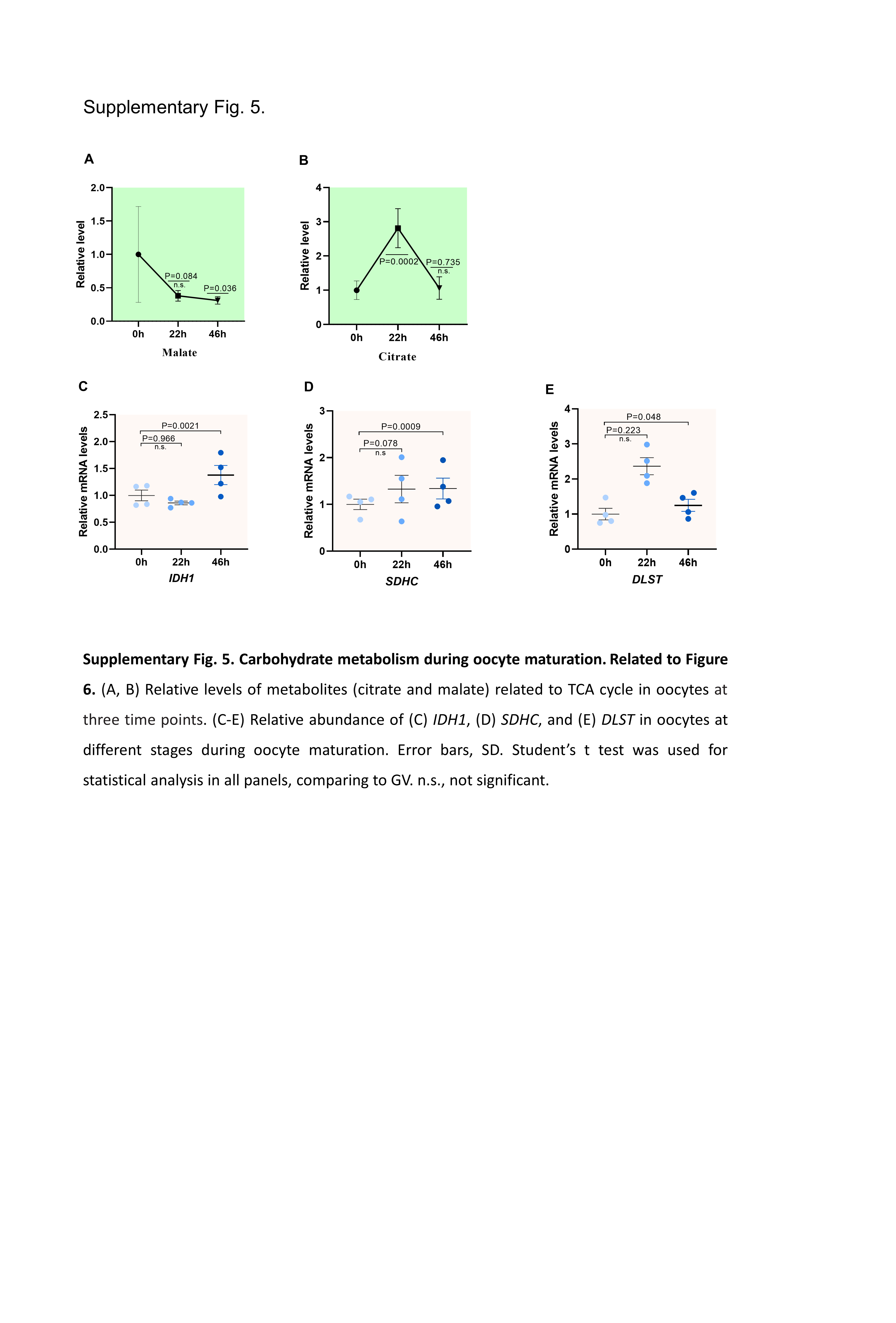

Supplement: Supplementary file 7 [file Image_5.tif]

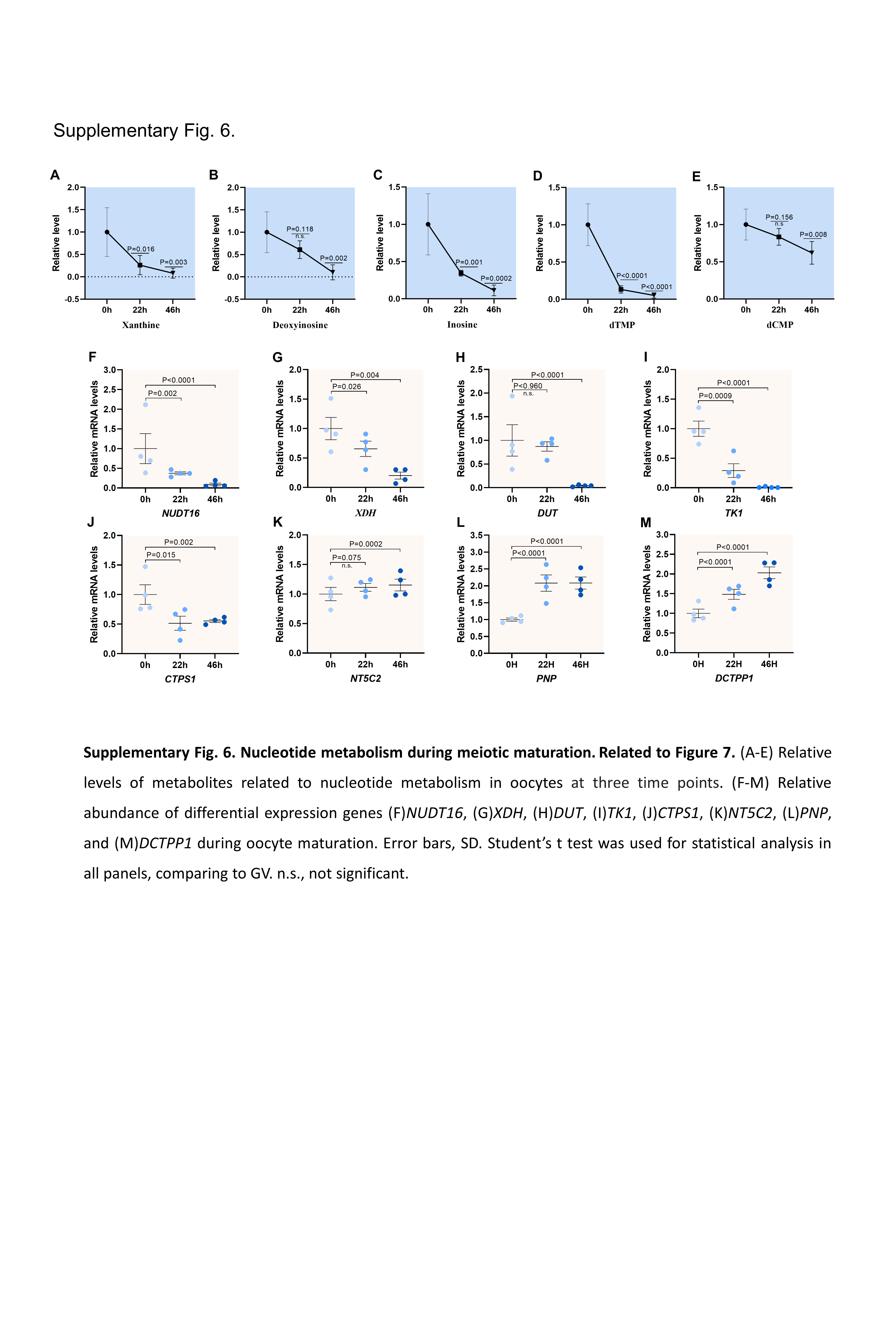

Supplement: Supplementary file 8 [file Image_6.tif]
